# *Rhodobacteraceae* are key players in microbiome assembly of the diatom *Asterionellopsis glacialis*

**DOI:** 10.1128/aem.00570-24

**Published:** 2024-05-29

**Authors:** Ashley Isaac, Amin R. Mohamed, Shady A. Amin

**Affiliations:** 1 Marine Microbiomics Lab, Biology Program, New York University Abu Dhabi, Abu Dhabi, United Arab Emirates; 2 Department of Molecular Ecology, Max Planck Institute for Marine Microbiology, Bremen, Germany; 3 Center for Genomics and Systems Biology, New York University Abu Dhabi, Abu Dhabi, United Arab Emirates; 4 Mubadala ACCESS Center, New York University Abu Dhabi, Abu Dhabi, United Arab Emirates; Norwegian University of Life Sciences, Ås, Norway

**Keywords:** diatoms, microbiome, *Rhodobacteraceae*, microbiome assembly

## Abstract

The complex interactions between bacterioplankton and phytoplankton have prompted numerous studies that investigate phytoplankton microbiomes with the aim of characterizing beneficial or opportunistic taxa and elucidating core bacterial members. Oftentimes, this knowledge is garnered through 16S rRNA gene profiling of microbiomes from phytoplankton isolated across spatial and temporal scales, yet these studies do not offer insight into microbiome assembly and structuring. In this study, we aimed to identify taxa central to structuring and establishing the microbiome of the ubiquitous diatom *Asterionellopsis glacialis*. We introduced a diverse environmental bacterial community to *A. glacialis* in nutrient-rich or nutrient-poor media in a continuous dilution culture setup and profiled the bacterial community over 7 days. 16S rRNA amplicon sequencing showed that cyanobacteria (*Coleofasciculaceae*) and *Rhodobacteraceae* dominate the microbiome early on and maintain a persistent association throughout the experiment. Differential abundance, co-abundance networks, and differential association analyses revealed that specific members of the family *Rhodobacteraceae*, particularly *Sulfitobacter* amplicon sequence variants, become integral members in microbiome assembly. In the presence of the diatom, *Sulfitobacter* species and other *Rhodobacteraceae* developed positive associations with taxa that are typically in high abundance in marine ecosystems (*Pelagibacter* and *Synechococcus*), leading to restructuring of the microbiome compared to diatom-free controls. These positive associations developed predominantly under oligotrophic conditions, highlighting the importance of investigating phytoplankton microbiomes in as close to natural conditions as possible to avoid biases that develop under routine laboratory conditions. These findings offer further insight into phytoplankton–bacteria interactions and illustrate the importance of *Rhodobacteraceae*, not merely as phytoplankton symbionts but as key taxa involved in microbiome assembly.

**IMPORTANCE:**

Most, if not all, microeukaryotic organisms harbor an associated microbial community, termed the microbiome. The microscale interactions that occur between these partners have global-scale consequences, influencing marine primary productivity, carbon cycling, and harmful algal blooms to name but a few. Over the last decade, there has been a growing interest in the study of phytoplankton microbiomes, particularly within the context of bloom dynamics. However, long-standing questions remain regarding the process of phytoplankton microbiome assembly. The significance of our research is to tease apart the mechanism of microbiome assembly with a particular focus on identifying bacterial taxa, which may not merely be symbionts but architects of the phytoplankton microbiome. Our results strengthen the understanding of the ecological mechanisms that underpin phytoplankton–bacteria interactions in order to accurately predict marine ecosystem responses to environmental perturbations.

## INTRODUCTION

As the dominant photosynthetic organisms in the ocean, phytoplankton play a pivotal role in generating oxygen and sequestering carbon via photosynthesis (1, 2), while serving as the primary trophic base for marine food webs (3, 4). Phytoplankton and bacteria have established intricate interactions such as commensalism, synergism, antagonism, parasitism, and competition over millions of years, contributing significantly to nutrient cycling and biomass production in the marine ecosystem (5, 6). Thus, the interplay between phytoplankton and their microbial associates is a fundamental and multifaceted ecological relationship in aquatic environments (4, 7). These interactions are largely regulated by phytoplankton-derived organic matter, which is essential for the growth and metabolism of both phytoplankton and bacteria. Bacteria rely on dissolved organic matter (DOM) secreted by phytoplankton to meet their nutritional demands (8), while the importance of heterotrophic bacteria in supporting the growth of phytoplankton through the production of diverse co-factors cannot be overemphasized. Additionally, some bacteria consume dead and senescent phytoplankton, which make up marine snow or particulate organic matter (POM) (9), leading to significant contributions to the carbon, nitrogen, and silicon biogeochemical cycles (10). The consumption of DOM and POM by bacteria, thus, facilitates the remineralization of organic matter fixed by phytoplankton back into inorganic components, which is a crucial process for the growth of phytoplankton and for global biogeochemical cycles (11, 12).

Phytoplankton and bacteria are intricately linked through chemical gradients within the phycosphere, a region around phytoplankton cells analogous to the plant root rhizosphere (6). The phycosphere facilitates the attraction of specific bacterial taxa with chemotactic abilities, ultimately leading to the formation of a symbiotic bacterial community around phytoplankton cells (13). These resulting bacterial assemblages are free-living and/or attached and are collectively termed the microbiome. The microbiome has been shown to exhibit variation in composition and dynamics across space and time (14) and during different life history stages of the host phytoplankton (15). Additionally, factors such as temperature, salinity, inorganic nutrient availability, and grazing may contribute to shaping the microbiome (16–19). The structuring of microbial communities is primarily explained by two main theories: (i) the neutral theory, which posits that all species are equivalent and colonization is a stochastic process (20, 21), and (ii) the niche theory, which suggests that interspecies competition and taxon-specific traits shape the community structure (22, 23). Dumbrell et al. (24) proposed a combinatorial theory, the lottery hypothesis (25, 26), which posited that colonization of the available niche space occurs randomly from a pool of functionally equivalent co-existing species. Kimbrel et al. (27) demonstrated that the microbiome assembly is influenced by the phytoplankton host, culture conditions, and initial inoculum composition, but their findings were inconclusive as they compared communities assembled *via* laboratory enrichment to outdoor mesocosms that had differing chemical environments. Conversely, Mönnich et al. (28) provided strong evidence against the lottery assembly model in favor of niche-based assembly, revealing convergent assembly of the microbiome of the diatom *Thalassiosira rotula* when co-cultured with different bacterial inocula.

These studies typically employ 16S rRNA gene profiling of bacterial communities to provide insight into microbial community diversity. In the case of diatom microbiomes, such analysis has identified consistent associations between specific diatom taxa and bacterial species, while others have revealed changes in the composition and structure of the bacterial community with time and location for a given diatom species. Bacterial taxa that have been identified to interact with diatoms and phytoplankton in general tend to belong to specific members of the *Proteobacteria* and *Bacteroidetes* phyla, including the *Sulfitobacter*, *Pseudosulfitobacter*, *Roseobacter*, *Alteromonas*, and *Flavobacterium* genera (5, 10, 29, 30). Most microbiome studies, however, tend to follow the microbial community associated with an “aging host population,” in what can be described as a batch culture. Typically, a bacterial inoculum is introduced to the host and samples are collected after a number of days (4–7 days) effectively taking a temporal snapshot of the microbiome, which does not offer insights into community structuring and dynamics. To address this limitation, other methods such as dilution cultivation and chemostat systems could be employed. These methods have proven effective for isolating low-abundance taxa (31) and studying microbiome community structures (32, 33), which together could help elucidate diatom microbiome assembly.

In this study, we investigate the microbiome of the diatom *Asterionellopsis glacialis* using a simple continuous dilution culture to maintain a constant cell density and culture volume. Notably, this approach keeps the diatom cell density constant and the population actively growing at the mid-log phase and, in so doing, follows the development and community structure of the microbiome over time without significant changes in nutrient bioavailability, which would influence the microbiome composition and diatom physiology. Thus, rather than reporting on the typical microbiome composition and diversity, we aim to utilize co-abundance and differential networks to identify key bacterial taxa that are involved in shaping the overall microbiome of *A. glacialis*.

## MATERIALS AND METHODS

### Preparation of diatom cultures and bacterial inoculum

Axenic *A. glacialis* A3 (CCMP3542) cultures were prepared and maintained as previously described (29, 34). All diatom cultures were maintained in F/2 media at 22°C in a 12:12 h light/dark diurnal cycle (125 µE m^−2^ s^−1^). Filtered seawater alone was confirmed to maintain *A. glacialis* A3 growth over a 24 h period at approximately one division per day (Fig. S1). The cultures were regularly checked for axenicity by nucleic acid staining with SYBR Safe Stain (Edvotek Corp, USA) under fluorescence microscopy. Axenicity was also confirmed by inoculation of the diatom culture into marine broth and checking for bacterial growth and contamination after 48 h. In preparation for co-culture experiments, axenic *A. glacialis* A3 was grown to the mid-exponential phase. Diatom growth was estimated using *in vivo* fluorescence of chlorophyll *a* (10-AU Fluorometer, Turner Designs, San Jose, CA, USA) as described previously (34).

**Fig 1:**
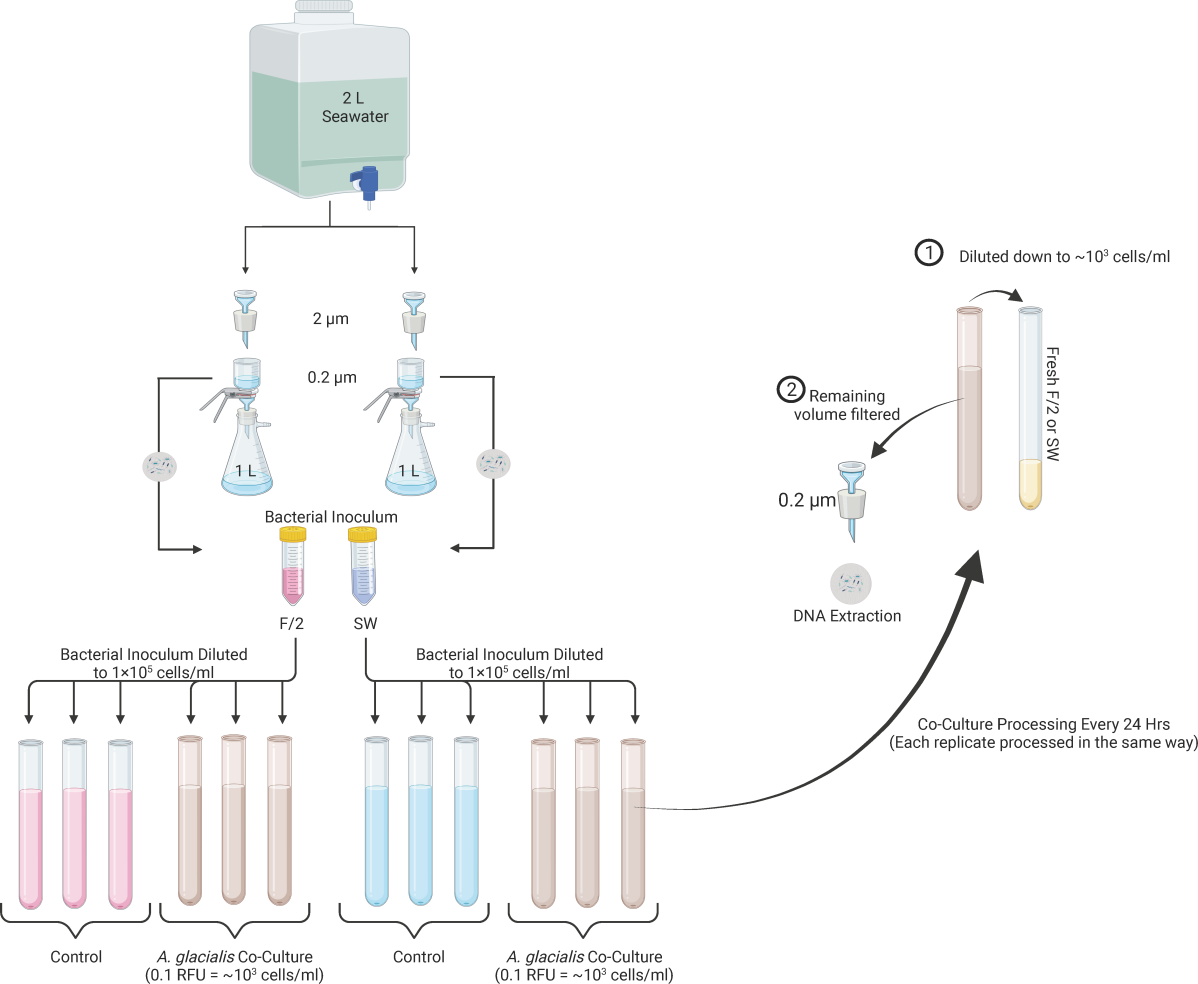
Water sample processing, experimental setup, and downstream analysis. Twenty-five liters of surface seawater was collected and processed. One liter (×2) was sequentially filtered with the 0.2 µm not allowed to run dry and resuspended in either F/2 or SW, which served as the inoculum for co-culture experiments. Additional 0.2 µm filters were processed and stored for DNA extraction and sequencing to provide information on the original (day 0) microbial community; refer to Materials and Methods for full details. Exponentially growing axenic *A. glacialis* A3 was diluted to 0.1 relative fluorescence units (RFU), ~10^3^ diatom cells/mL in either SW or F/2 medium in preparation for co-culture with bacterial inocula. The bacterial inocula was introduced to diluted diatom culture to a final concentration of ~1 × 10^5^ cells/mL. Controls consisted of bacterial inocula and no diatoms. The triplicate SW and F/2 co-cultures and controls were incubated for 7 days. Every 24 h, diatom growth was measured in the co-cultures. The volume required to dilute the culture back down to ~10^3^ diatom cells/mL was retained in the tube, and the remainder was removed and filtered onto a 0.2 µm membrane filter for DNA extraction. The volume retained in the original tube was brought back up to 25 mL with either sterile SW or F/2. Each day, the controls were treated in a similar fashion. As dilution volumes were calculated based on diatom cell density, the volumes to filter and retain for the controls were based on the mean volumes used in the co-cultures every 24 h for the respective media. The 0.2 µm filters were stored for DNA extraction.

Approximately 25 L of surface seawater was collected off the coast of Abu Dhabi (24° 38′16.0″N 54° 27′43.1″E), transported back to the laboratory, and processed within 2 h of collection. Fifteen liters of the collected seawater was prefiltered through a 100 µm mesh and distributed equally between three carboys. Each 5 L replicate was sequentially filtered through 10, 2, and 0.2 µm polycarbonate membrane filters (10 and 0.2 µm: Whatman, UK; 2 µm: Isopore, Germany). The 0.2 µm filters were flash-frozen and stored for DNA extraction to later examine the inoculum community. Another 5 L of seawater was sequentially filtered through 10, 2, and 0.1 µm filters (in an attempt to ensure sterility by the removal of potential mycoplasmas) to be used for co-culture experiments (this 0.1 µm filtered seawater will be referred to as SW). Finally, 1 L (×2) of seawater was filtered sequentially through 2 µm and concentrated onto 0.2 µm filters to obtain the bacterial inoculum (starter community) for the experiment (Fig. 1). To increase the viability of cells, filters were prevented from running dry during this process. The concentrated cell suspension on the 0.2 µm filters was diluted and resuspended in sterile 20 mL SW or sterile F/2 medium, and cells were counted via the CyFlow Space flow cytometer (Partec, Münster, Germany). This concentrated bacterial stock served as the inoculum for co-culture experiments.

### Co-culture experiments

Seawater was collected, and bacterial inocula were prepared once the axenic *A. glacialis* culture was at mid-exponential growth. The diatom culture was diluted to 0.1 RFU, approximately, 10^3^ cells/mL in either SW or F/2 medium (Fig. 1). The bacterial inocula prepared in either SW or F/2 were introduced to the newly diluted diatom culture to a final concentration of ~1 × 10^5^ cells/mL in a final volume of 25 mL. Bacterial community controls (BCCs) in which diatoms were not included were also set up for each media type. The triplicate SW and F/2 co-cultures and controls were incubated for 7 days, as described above. Every 24 h period over the 7-day experiment, diatom growth was measured in the co-cultures, and bacterial cells were counted via flow cytometry. Every 24 h, the volume required to dilute the culture back down to ~10^3^ diatom cells/mL was retained in the tube, and the remainder was removed and filtered onto a 0.2 µm membrane filter (Whatman, UK) and immediately flash-frozen and stored at −80°C until DNA extraction. The volume retained in the original tube was brought back up to 25 mL with either sterile SW or F/2. Each day, the BCC was treated in a similar fashion. As dilution volumes were calculated based on diatom cell density, the volumes to filter and retain for the BCC were based on the mean volumes used in the co-cultures every 24 h for the respective media. The 0.2 µm BCC filters were flash-frozen and stored at −80°C until DNA extraction.

### DNA extraction, sequencing, and bioinformatic analyses

Microbial genomic DNA from the filters were isolated using the DNeasy Power Water Kit (Qiagen) according to the manufacturer’s instructions. The V3-V4 region of the 16S rRNA gene was amplified and sequenced at NovogeneAIT Genomics (Singapore). Paired-end (2 × 250 bp) sequencing on the Illumina NovaSeq 6000 (San Diego, CA) platform was conducted using the primers 341F (5′-CCTAYGGGRBGCASCAG-3′) and 806R (5′-GGACTACHVGGGTWTCTAAT-3′).

Amplicon sequence variants (ASVs) were generated from clean raw reads using the rANOMALY package (v.1.0.0) (35) implementing DADA2 (36). Taxonomic classification was based on the SILVA v138 database. Alpha-diversity was assessed using observed ASVs and Shannon and Simpson diversity indices to investigate species richness and evenness. Beta-diversity was assessed and visualized by principal coordinate analysis (PCoA) based on pairwise Bray–Curtis distance and tested by the permutational analysis of variance (PERMANOVA). The differential abundance of taxa across individual days between co-culture and control samples was identified with metagenomeSeq from the microbiomeMarker R package (v.1.2.2) (37) with a *P*-adjusted value cutoff of <0.05. All plots were generated using the ggplot2 (v.3.4.2) (38) and phylosmith (v.1.0.6) (39) R packages, and all statistical analyses were performed on RStudio 2022.07.1 Build 554.

Microbial association networks were inferred, and plots were generated with the NetCoMi R package (v.1.1.0) (40). Initial association networks were constructed using the Sparse Inverse Covariance Estimation for Ecological Association Inference method using default parameters of the *netConstruct* function (soft-thresholding: 0.8) (41), which has been designed to handle compositional data and assumes a sparsely connected underlying network. ASVs or nodes of importance were identified based on their eigenvector centrality measures. This measure provides an indication of the centrality of a node based on the centrality of its neighbors; thus, taxa with high eigenvector centrality are likely to be central to the network as a whole (42, 43). Control and co-culture networks were compared against each other quantitatively by calculating their Jaccard index, which indicates similarity between sets of the most central nodes (Jacc = 0, lowest similarity and Jacc = 1, highest similarity), and the adjusted Rand index (ARI), which indicates the dissimilarity between clustering of nodes (ARI = 1: identical clustering and ARI = 0: dissimilar clustering) (40). We thereafter sought to uncover differential associations between the control and co-culture networks. Sparse networks do not allow for this, and thus, networks based on Spearman correlations were created, and significantly differentially associated taxa were identified by comparing the correlation coefficients with Fisher’s *z*-test (40). This was carried out using the *diffnet* function with default settings [false discovery rate parameter (*lfdr*) for multiple testing correction default was ≤0.2]. Nodes identified to have differential correlations were subsequently used to construct association networks of ASVs associated with these nodes.

SSU rRNA gene sequences were obtained from SILVA (44) and NCBI, which were closely related to ASVs of particular interest, specifically *Sulfitobacter* spp., which are known diatom and phytoplankton symbionts. The symbionts were as follows: *Ruegeria pomeroyi* DSS-3 (45), *Sulfitobacter* sp. SA11 (46), *Pseudosulfitobacter pseudonitzschia*e SMR1 (47), *Pseudosulfitobacter pseudonitzschiae* F5 (29, 30), *Sulfitobacter brevis*, *Sulfitobacter noctilucicola*, *Sulfitobacter pontiacus,* and *Sulfitobacter litoralis* (48). The marine opportunist *Alteromonas macleodii* (49) served as the outgroup. The sequences were analyzed in Geneious Prime (version 11.1.5). They were aligned with MAFFT, and a consensus maximum likelihood tree was constructed with RAxML through a rapid bootstrap approach using 100 replicates. The consensus tree was visualized in Geneious Prime.

## RESULTS

### Distinct microbial community assembly in F/2 and SW

The bacterial fraction of both co-cultures and controls in both types of media experienced a 24 h lag phase, and cell numbers sharply increased after 48 h (Fig. S2). The daily dilution of each incubation to maintain a constant diatom cell density resulted in bacterial cell densities remaining relatively stable (~4.4 × 10^5^–1.2 × 10^6^ cells/mL depending on the treatment) across the rest of the incubation period (days 2–7). Higher bacterial cell densities were observed in the SW co-cultures compared to the control and F2 incubations (Days 3–5) followed by a decline over days 6–7 (Fig. S2). The F/2 incubations remained relatively stable from day 2 onward with no significant difference between the co-culture and control groups. Diversity metrics across F/2 and SW incubations indicate that there was an overall decrease in α-diversity (Shannon and Simpson) in co-cultures and controls compared to the day 0 inoculum (Wilcox, *P* < 0.01 and *P* < 0.05 for Shannon and Simpson, respectively) (Fig. S3a). Additionally, species richness (number of observed ASVs) was significantly lower in SW and F/2 compared to the inoculum (Wilcox, *P* < 0.01). In addition, significant differences were observed between the diversity in F/2 and SW (PERMANOVA: *P* < 0.001), with the initial inoculum displaying a diversity lying in between both media (Fig. S3b).

### *Rhodobacteraceae* and cyanobacteria respond to the presence of diatoms

**Fig 2:**
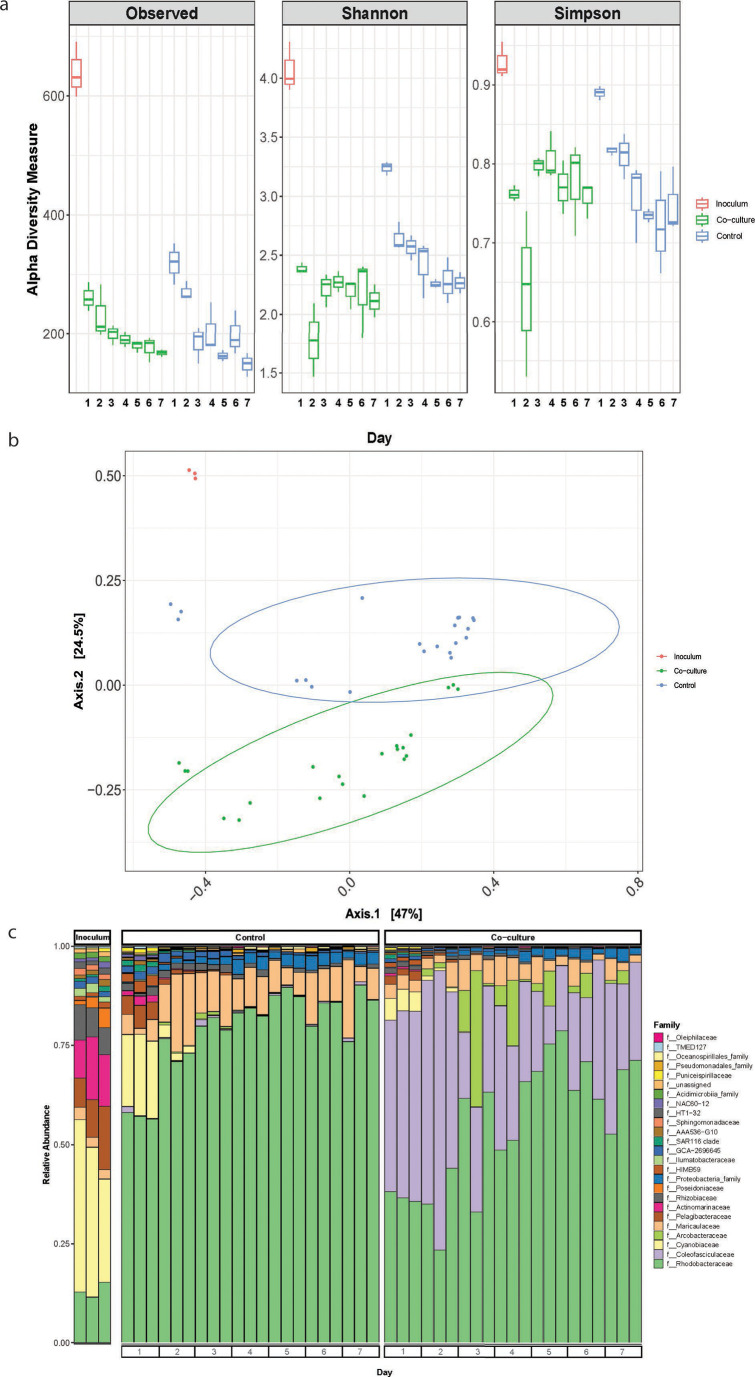
Microbiome diversity and composition in F/2 incubations over 7 days. (a) Alpha-diversity indices of observed ASVs and Shannon and Simpson of starter inoculum culture and across controls and co-cultures for each day. The first box plot (red box) for each metric represents the starter inoculum. (b) PCoA of Bray–Curtis distances between starter inocula, controls, and co-culture incubations (PERMANOVA, *P* < 0.001). (c) Relative abundance of the top 25 microbial families based on 16S rRNA amplicon sequencing of the initial inoculum, control, and co-culture set-ups over a 7-day incubation period. All analyses are based on three biological replicates.

**Fig 3:**
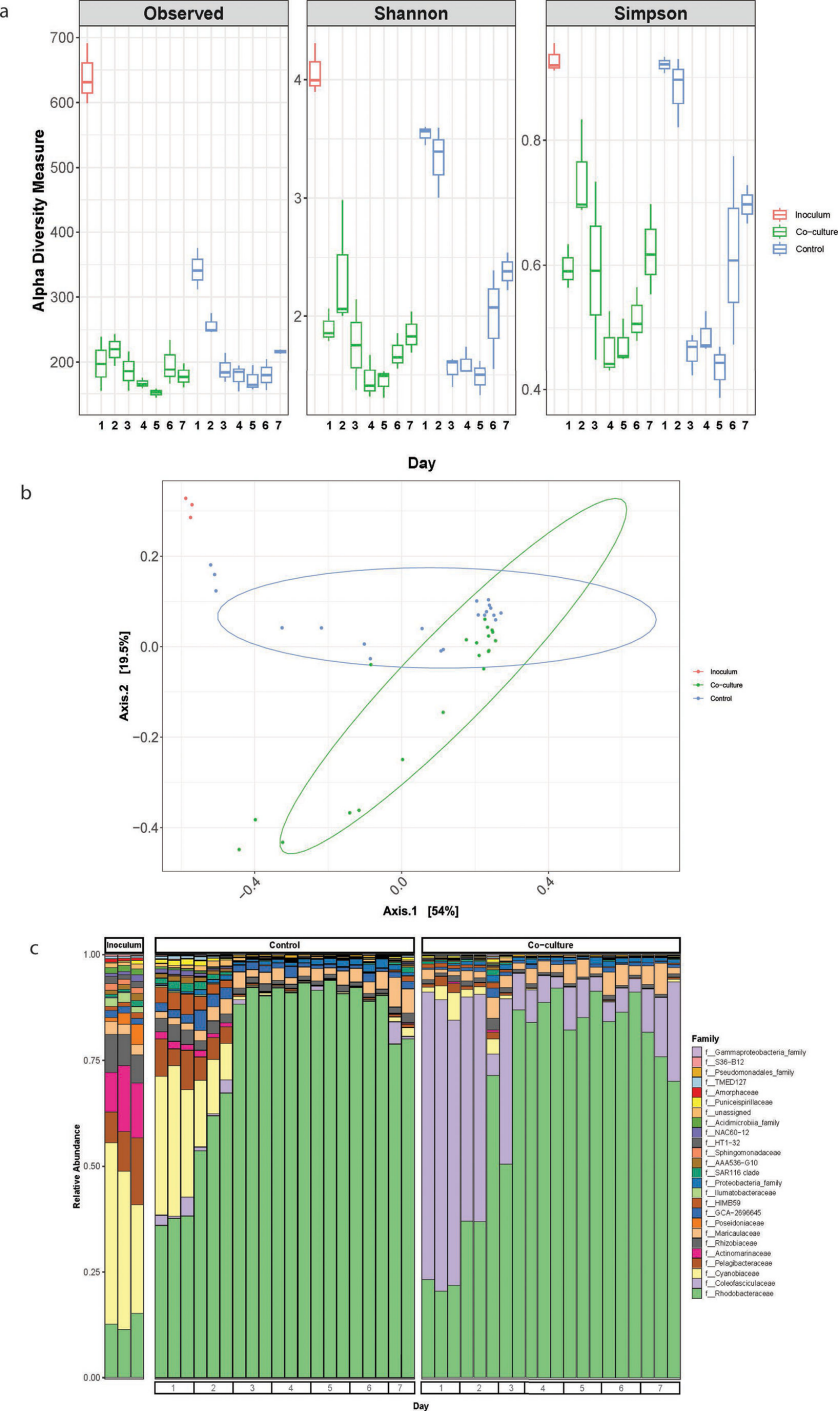
Microbiome diversity and composition in SW incubations over 7 days. (a) Alpha-diversity indices of observed ASVs and Shannon and Simpson of starter inoculum culture and across controls and co-cultures for each day. The first box plot (red box) for each metric represents the starter inoculum. (b) PCoA of Bray–Curtis distances between starter inocula, controls, and co-culture incubations (PERMANOVA, *P* < 0.001). (c) Relative abundance of the top 25 microbial families based on 16S rRNA amplicon sequencing of the initial inoculum, control, and co-culture set-ups over a 7-day incubation period. All analyses are based on three biological replicates except for day 7 in the control and day 3 of the co-culture as these samples failed sequencing quality control.

Diversity metrics in the F/2 incubations reveal that species richness and evenness were lower in co-cultures than in controls, though not significantly so (Fig. 2a). Beta-diversity across the entire incubation period shows the presence of distinct microbial communities in the co-cultures compared to controls (PERMANOVA: *P* < 0.001) (Fig. 2b). Similar α- and β-diversity trends were observed in SW incubations. Species richness and evenness were once again lower in co-culture than in controls without statistical significance; however, there was a slight increase in α-diversity metrics on days 6–7 that coincides with the decrease in bacterial cell counts for the SW incubations (Fig. 3a). Different microbial communities were observed in the co-culture and the control (PERMANOVA: *P* < 0.001); however, the communities appear to converge toward the end of the incubation period (Fig. 3b). The taxonomic profiles over the course of 7 days in both F/2 and SW were diverse and presented interesting trends (Fig. 2c and 3c). In F/2 media, *Maricaulaceae* had a stable and constant presence in the co-culture but had a higher abundance from day 2 in the control. There was a brief spike in the abundance of *Arcobacteraceae* at day 3 in the co-culture, but it was almost non-existent in the control. In SW media, *Pelagibacteraceae* and *Actinomarinaceae* had higher abundance on days 1 and 2 of the control relative to the co-culture, while *Maricaulaceae* had a stable presence in both co-culture and controls but lower than that in F/2 incubations.

The most interesting observation was that *Rhodobacteraceae* [recently renamed *Roseobacteraceae* (50)] performed extremely well under laboratory conditions, and their relative abundance increased considerably in both co-culture and control for both types of media. There were 723 *Rhodobacteraceae* ASVs identified across the entire data set; however, there was little overlap of shared ASVs across different media types, as well as their respective controls. F/2 and SW incubations contained 439 and 373 *Rhodobacteraceae* ASVs, respectively, of which 152 ASVs were common in both media (21% of all identified *Rhodobacteraceae*). The F/2 control possessed 287 *Rhodobacteraceae* ASVs while the co-culture had 305, of which 153 were in common (21% of all identified *Rhodobacteraceae*), while the SW control had 237 *Rhodobacteraceae* ASVs and its respective co-culture had 258 of which 122 were in common (16.8% of all identified *Rhodobacteraceae*).

Another interesting trend was that the cyanobacterial family *Cyanobiaceae* (ASVs resolved at the species level as *Synechococcus*) persisted in co-cultures and controls in both F/2 and SW in days 1–2, though generally, their abundance declined relative to the initial inoculum. Interestingly, the relative abundance of *Cyanobiaceae* in co-cultures was significantly lower than in controls, suggesting potential competition with *A. glacialis* (Fig. 2c and 3c). Like *Rhodobacteraceae*, filamentous cyanobacteria belonging to the family *Coleofasciculaceae* increased significantly after the first 24 h in F/2 and SW co-cultures and continued to persist throughout the 7-day incubation.

### Interspecies interactions in SW are more complex

**Fig 4:**
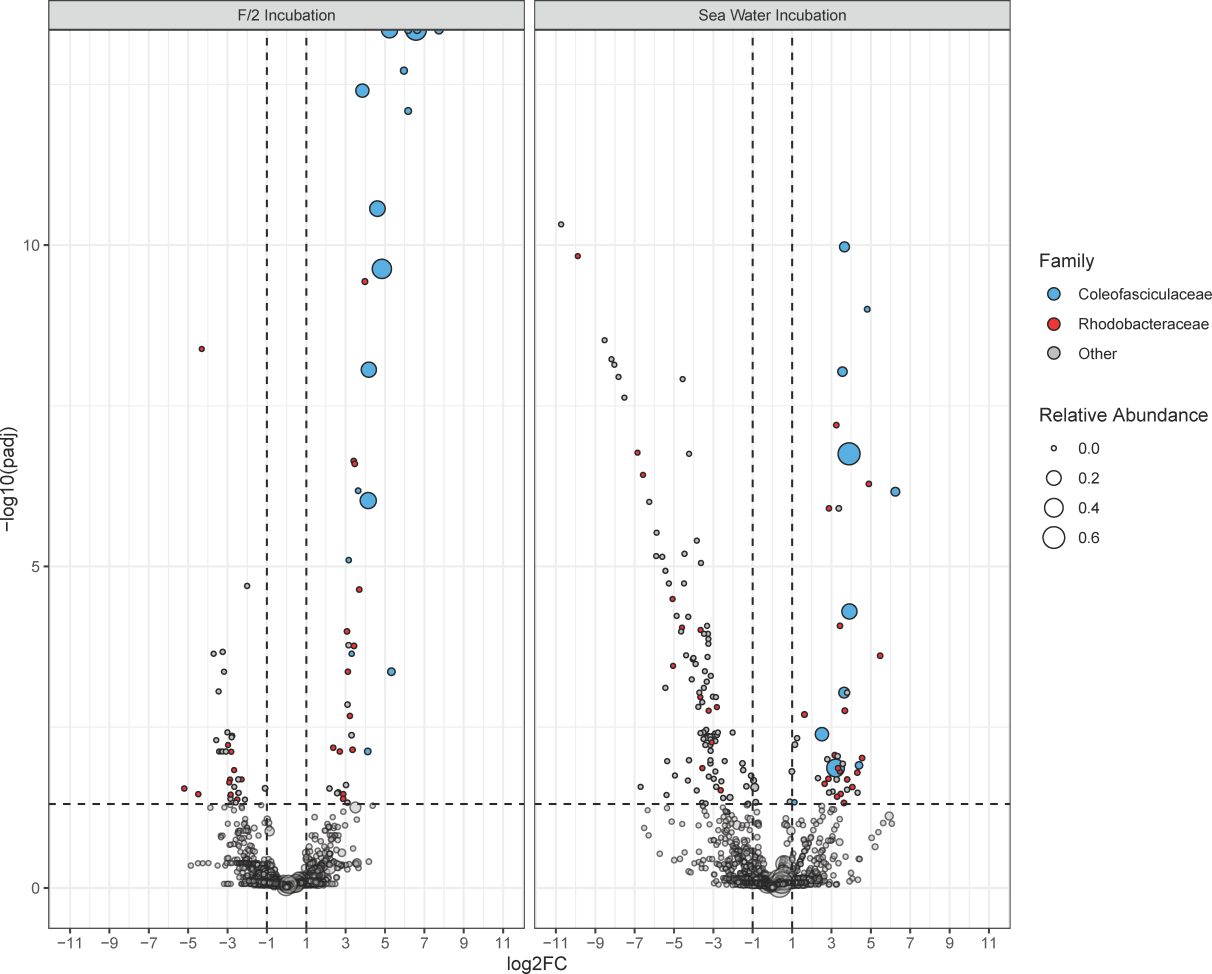
Volcano plot of the distribution of ASVs belonging to differentially abundant taxa under different culture conditions according to their log_2_-fold change and *P*-adjusted value. MetagenomeSeq was used to calculate log_2_-fold change and *P*-adjusted values of ASVs in co-culture incubations, relative to the controls for each respective day and respective medium. ASVs with *P*-adjusted <0.05 were considered significantly differentially abundant; hence, points below the horizontal dashed line are not significantly differentially abundant. Points are colored based on taxonomic family, and the size of the point represents the average relative abundance for the day in which the ASV was determined to be differentially abundant. See Table S1 for the list of differentially abundant ASVs.

We performed differential abundance analysis with metagenomeSeq (*P*-adjusted <0.05) to identify specific ASVs that had a significant increase or decrease in their relative abundance in the co-cultures relative to their respective controls. Cumulatively, across the individual days, 71 and 194 ASVs were identified as differentially abundant in F/2 and SW incubations, respectively (Fig. 4; Tables S1 to S3), and were reflective of the taxonomic trends described above. ASVs belonging to the families *Coleofasciculaceae* and *Rhodobacteraceae* were among the most differentially abundant across both types of media; however, there was little overlap of specific differentially abundant ASVs between the two media types (Fig. 4; Table S3). Over the 7-day incubation, *Coleofasciculaceae* accounted for 44.7% and 23.9% of differentially abundant ASVs in F/2 and SW co-cultures, respectively (Table S3). ASVs displaying decreased differential abundance included cyanobacterial ASVs belonging to *Cyanobiaceae* that accounted for 3% and 12.1% of differentially decreased ASVs in F/2 and SW, respectively (Table S3). In addition, *Pelagibacteraceae* accounted for 3% and 9.5% of differentially decreased ASVs in F/2 and SW, respectively (Table S3). Interestingly, *Rhodobacteraceae* ASVs displayed contrasting patterns of some ASVs displaying increased differential abundance at 36.8% and 41.3% in F/2 and SW, respectively, while others accounted for 36% and 16% of decreased differential abundant ASVs in F/2 and SW, respectively (Tables S1 and S3). Overall, the most striking feature was that 76.3% of differentially abundant ASVs in SW co-cultures showed a decreased abundance relative to controls, in contrast to 46.5% in F/2 (Tables S2 and S3).

Given that *Coleofasciculaceae* and *Rhodobacteraceae* had such a high prevalence in both controls and co-cultures and were among the ASVs that were most differentially abundant, we sought to identify specific ASVs involved in structuring the overall architecture of the microbiome. To do so, we created sparse networks based on eigenvector centrality to identify nodes that are likely central to the network as a whole. At first glance, the co-culture and control networks look strikingly similar for the respective media, and global features confirm this perceived similarity (Fig. S4 and S5; Data S1). Quantitative network analysis, however, revealed significant differences between co-culture and control networks. Specific nodes with high eigenvector centrality were identified as hubs. The top five ASVs with the highest normalized eigenvector centrality for F/2 and SW incubations are provided in Data S2 and S3. *Rhodobacteraceae* hubs were more prevalent in F/2 co-cultures than SW (Data S2 and S3). In contrast to F/2, though present, the hub taxonomic profile in SW was not dominated by *Rhodobacteraceae* and instead had a diverse array of taxa belonging to families such as *Pelagibacteraceae*, *Alteromonadaceae*, *Cyanobiaceae*, *Rhizobiaceae,* and *Actinomarinaceae* (Data S3).

Jaccard indices were calculated to test for similarities in network centrality measures of the co-culture and control networks. Centralities such as degree, closeness, betweenness, and eigenvector centrality were all significantly different, as shown by a low degree of similarity between co-cultures and controls of the respective media (Data S2 and S3). Hub taxa in co-culture networks were significantly different from hub taxa in control networks for both F/2 (Jaccard index = 0.062, *P* < 0.001) and SW (Jaccard index = 0.1, *P* < 0.01) incubations. The adjusted Rand indices confirm the low degree of similarity between the networks as indicated by only low to moderate clustering similarities between controls and co-cultures (F/2 ARI = 0.105, *P* = 0; SW ARI = 0.071, *P* = 0).

### *Rhodobacteraceae* are central to diatom microbiome assembly

Given the dissimilarity in network topologies and differing hub taxa, we sought to uncover differential associations among ASVs between the co-cultures and control networks. Sparse networks do not allow for this, and thus, networks based on Spearman correlations were created, taking all pairwise associations into consideration. There were 11 and 47 differentially correlated nodes between co-culture and control networks in F/2 and SW networks, respectively (Fig. S6 and S7). Of particular note was the development of many more positive associations in SW co-culture incubations compared to F/2 co-culture incubations. There were sparse differential associations in F/2 incubations. A single *Pelagibacter* ASV (Pelagibacter_species4) shifted from positive to negative associations with *Cyanobiaceae* (RCC307_sp0000635251 and SynechococcuE_species2), while a *Coleofasciculaceae* member (SIO2C1_sp0106729251) developed a positive interaction with *Cyanobiaceae* (Cyanobiaceae_species1) in the co-culture experiment. Additionally, two *Rhodobacteraceae* ASVs (Shimia_species5 and MED−G52_species1) changed from negative to positive associations in the co-culture (Fig. S6). Given the very limited number of differential associations in F/2, strong conclusions are unlikely to be drawn from these incubations. However, the differential associations uncovered in the SW incubations were more informative. Once again, the same *Pelagibacter* (Pelagibacter_species4) ASV proved to be an important member in the SW differential network, forming multiple strong positive associations in the co-culture with multiple taxa, but not in the control. The same was true for a member of the family *Cyanobiaceae* [RCC307_sp0000635251 (*Synechococcus* sp. RCC307)] (Fig. S7). Many of these putative “keystone” species in the co-culture belong to the family *Rhodobacteracae*, particularly members of the genus *Sulfitobacter* (Fig. S7).

**Fig 5:**
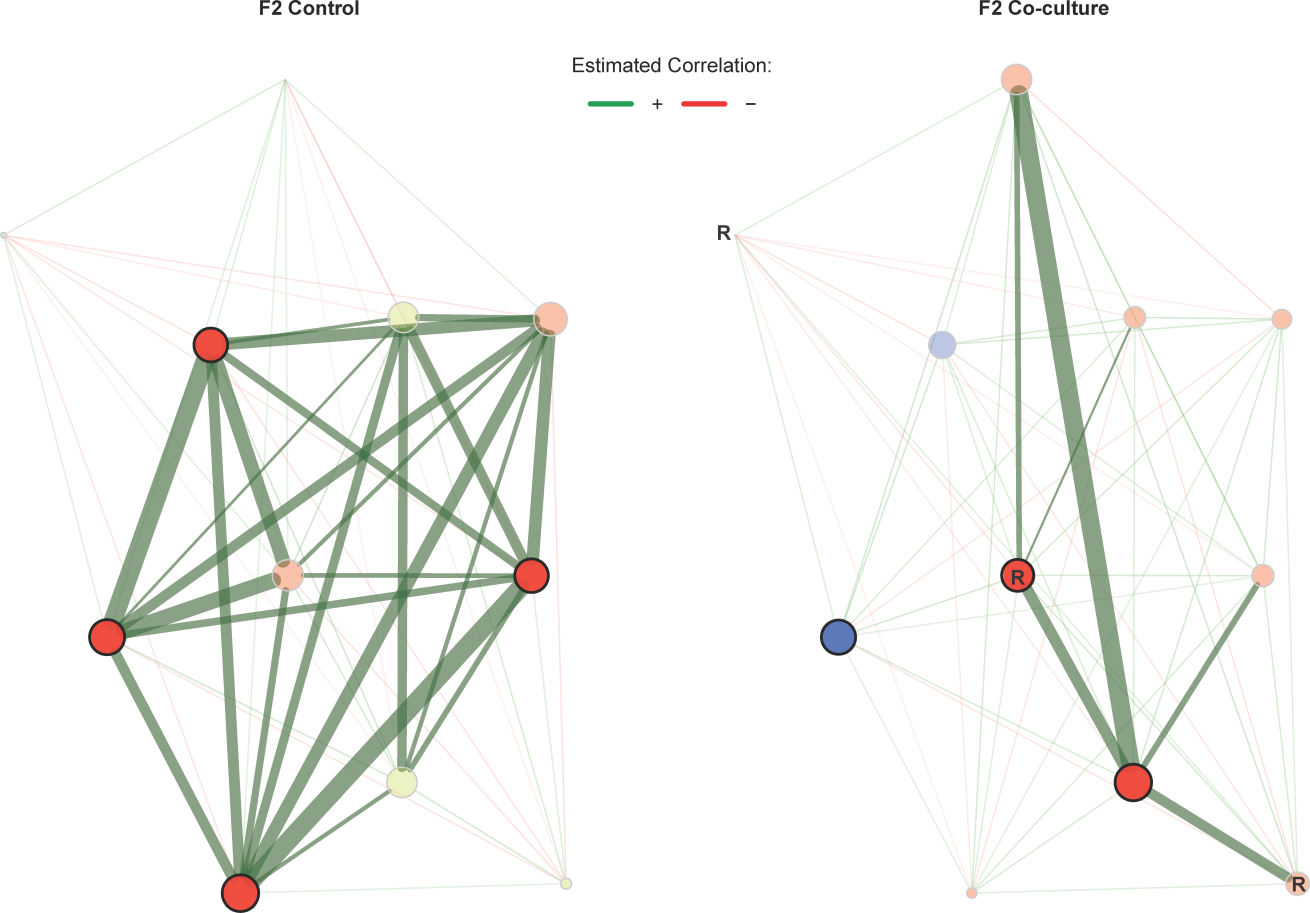
Comparison of co-occurrence networks based on differentially connected bacterial taxa in control and co-culture incubations in F/2 media. Nodes represent individual ASVs that were identified to be differentially correlated in Fig. S6. Edges represent either positive (green) or negative (red) associations. Nodes that share the same color form clusters that are more connected relative to other nodes. The size of the node is relative to the eigenvector centrality, i.e., the larger the node, the more central that node is to the network as a whole. Nodes that are less transparent and that have dark outlines have been identified as hubs, based on their eigenvector centrality. Edge width represents the strength of the association with thicker lines indicating stronger associations (applied to positive associations only given the trend observed in differential networks). Nodes indicated with an **R** (within or adjacent to the node) belong to the family *Rhodobacteraceae*.

**Fig 6:**
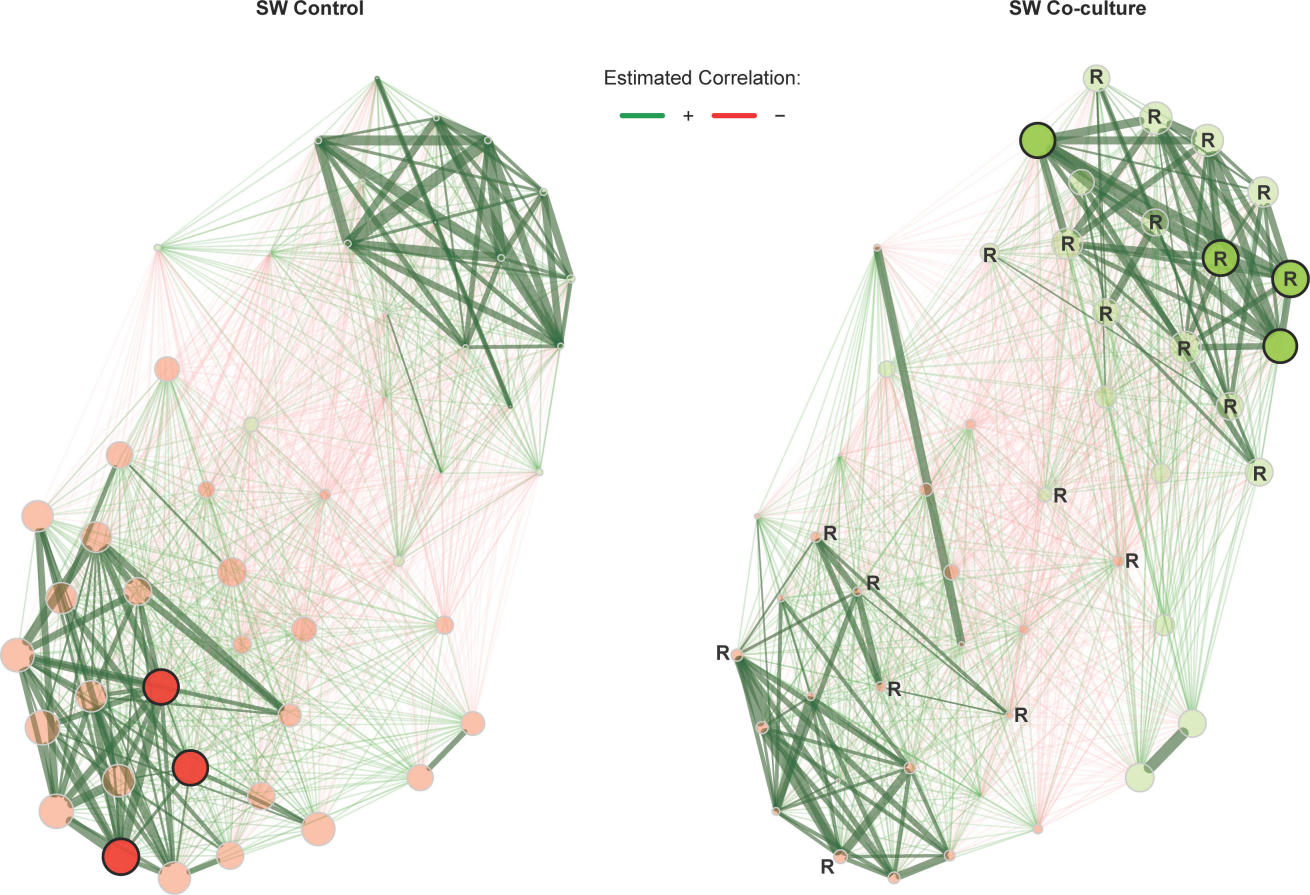
Comparison of co-occurrence networks based on differentially connected bacterial taxa in control and co-culture incubations in SW media. Nodes represent individual ASVs that were identified to be differentially correlated in Fig. S7. Edges represent either positive (green) or negative (red) associations. Nodes that share the same color form clusters that are more connected relative to other nodes. The size of the node is relative to the eigenvector centrality, i.e., the larger the node, the more central that node is to the network as a whole. Nodes that are less transparent and that have dark outlines have been identified as hubs, based on their eigenvector centrality. Edge width represents the strength of the association with thicker lines indicating stronger associations (applied to positive associations only given the trend observed in differential networks). Nodes indicated with an **R** (within or adjacent to the node) belong to the family *Rhodobacteracea*

Association networks were subsequently constructed using the differentially associated ASVs. The microbiome interactions in F/2 co-culture incubation were drastically reduced compared to the control (Fig. 5). However, there were still distinct changes in the co-culture system with an overall weakening of positive associations and taxa switching to different clusters. In contrast, there was a pronounced effect on SW co-cultures. There were many more positive associations that developed in the SW co-culture incubation compared to the control, with many of these interactions occurring between ASVs from the family *Rhodobacteracae* (Fig. 6; Fig. S7). The ASVs, Pelagibacter_species4 and *Synechococcus* sp. RCC307 (RCC307_sp0000635251), switched clusters in the co-culture (red to green in the lower right corner), associating more with members of the family *Rhodobacteracae*. The eigenvector centralities, indicating importance in the network, increased considerably for members of the family *Rhodobacteracae* [concentrated in the top right cluster indicated with R (Fig. 6)], and particularly, ASVs from the genus *Sulfitobacter* became hub taxa in the co-culture network. Given the presumed importance of *Sulfitobacter* ASVs in the community, we sought to determine if they were closely related to *Sulfitobacter* spp. that are known symbionts of diatoms or other phytoplankton. A phylogenetic tree of the seven *Sulfitobacter* ASVs identified in the differential association network (Fig. S7) along with other *Sulfitobacter* spp. was constructed (Fig. S8). The majority of the *Sulfitobacter* ASVs of interest clustered closely to *S. pontiacus,* and one other ASV showed close relation to *S. noctilucicola*, both of which were identified as phytoplankton symbionts.

## DISCUSSION

It has been well established that microbiomes play an integral role in the development, health, and function of eukaryotic hosts. In the case of phytoplankton, devoid of a “body,” single phytoplankton cells serve as microenvironments for bacteria. Their interactions coalesce in the immediate space surrounding host cells, the phycosphere, a region analogous to the plant rhizosphere. The phytoplankton phycosphere is effectively colonized by free-living and attached bacterial associates, which are collectively considered as the microbiome. There are many theories and ecological principles that aim to explain the assembly and diversity of microbial communities; however, researchers are yet to reach a consensus, with the two most hotly debated principles being deterministic processes and ecological stochasticity (51). For phytoplankton, it is accepted that both deterministic and stochastic processes influence the microbiome structure, though the relative contributions of each are still unclear. Much of the research of recent times focuses heavily on deterministic processes and particularly on host effects and selection (30, 52–57). An important factor missing to date is the effects and contributions to microbiome structuring by the selected microbes within that microbiome. Indeed, biotic interactions of this nature are difficult to unravel due to the immense diversity in microbial communities. A potential solution to this dilemma is by identifying keystone microbes that have cascading interactions through a given community (58). In this study, we suggest that the first stages toward colonization are the selection and subsequent restructuring of the microbial community by specific members of early colonizers.

### Culture media and diatoms are microbiome influencers

In our experimental set-up, we hypothesized that the abundance of bacteria in co-cultures would be linked to phytoplankton growth. In contrast, bacterial abundance in control set-ups would rely solely on residual organic matter present in the seawater used to prepare F/2 and SW. The daily dilutions produce relatively stable bacterial cell densities over days 2–6 (Fig. S2) and reflect what might be observed in a chemostat. Bacterial growth and abundance are inherently linked to nutrient availability, and under favorable conditions in chemostat cultures of phytoplankton and zooplankton, they have been shown to influence population dynamics (59). It was interesting to note that SW alone was sufficient to maintain relatively high and stable bacterial cell densities (Fig. S2). This suggests that the bacteria that thrive in SW are unlikely to be limited by the absence of nutrients that are found in F/2. Additionally, our results show that the media type plays a major role in species diversity, regardless of the presence of the diatom (Fig. S3a and S3b). Earlier reports show that bacteria inoculated into seawater or seawater amended with nutrients typically result in rapid community shifts toward differing culturable genera (60). Sapp et al. (61) investigated the microbiome of four diatom species that developed after 2–4 months in F/2 and noted that the bacterial communities were significantly different from the *in situ* community (the bacterial community from seawater collected on the day of diatom isolation). Our results indicate that the presence/absence of nutrients is a major driving force in the establishment of a microbiome and may bias our understanding of microbiomes of lab-maintained phytoplankton cultures.

A second layer of bacterial community modulation is brought about by the presence of the diatom, which plays a significant role in the development of distinct microbial communities in co-cultures in both media relative to their respective controls (Fig. 2b and 3b). Phytoplankton may exude DOM in the form of polysaccharides both passively and actively (62), which are important factors that influence the heterotrophic bacterial community that associates with phytoplankton (63, 64). There is overwhelming evidence that supports the fact that microbiomes are unique among different diatom species and strains and consistent across time (14, 34). Additionally, free-living and attached members of the microbiome differ over the life history of the host (63). These observations are not unique to diatoms as there are similar reports for dinoflagellates and coccolithophores (65–68). These studies provide valuable information on taxa that may be core members of the microbiome of specific phytoplankton taxa; however, they do not provide context on their importance to the community as a whole. In this study, we show that different microbiomes develop when phytoplankton are cultivated and maintained under different nutrient concentrations and compositions (F/2 or SW). This highlights that the presumed importance of taxa that have routinely been identified as common associates of phytoplankton might be biased under laboratory conditions. In an attempt to gain a deeper understanding, subsequent analysis was conducted separately for SW and F/2 incubations, to identify taxa that are important in structuring the overall microbiome in the respective media.

### Identifying bacterial taxa of general importance in the diatom microbiome

The taxonomic profiles of the co-culture incubations in both F/2 and SW (Fig. 2c and 3c) indicate that *Rhodobacteraceae* had one of the most drastic responses to *in vitro* cultivation. Members of the *Rhodobacteraceae* have traditionally been classified as ecological generalists (69–71) and have been shown to grow at relatively high growth rates in the laboratory (72) as observed in the controls. However, *A. glacialis* is known to produce central (e.g., histidine, leucine, and citruline) and secondary (particularly azelaic acid and rosmarinic acid) metabolites that promote growth and attachment of several members of the *Rhodobacteraceae* (30). *Rhodobacteraceae* were recently shown to not simply be generalist in nature, but there are select members of this group that thrive and interact closely with phytoplankton (73). Wang et al. (74) confirm this by reporting that during phytoplankton blooms, specific *Rhodobacteraceae* genera are predominantly found attached to phytoplankton cells and particles, while other *Rhodobacteraceae* genera are predominantly free-living. Indeed, beneficial interactions between many members of the *Rhodobacteraceae* and different phytoplankton lineages have been reported (45, 46, 75). It is, therefore, likely that there is a subset of *Rhodobacteraceae,* which are more prone to interactions with diatoms than others.

Filamentous cyanobacteria, particularly members of the family *Coleofasciculaceae,* also appeared to thrive within the co-cultures. This is curious as *Coleofasciculaceae* was present at relatively low abundance in the inoculum (<0.1%) yet increased considerably on day 1, yet this did not translate to a linear increase in bacterial cell numbers on day 1. This could be a result of several long-standing factors that continue to plague microbiome studies; examples include PCR bias from amplicon sequencing, sequencing bias, or the presence of multiple 16S rRNA gene copies (76–78). Some filamentous cyanobacteria can fix nitrogen and are key members in the N_2_ cycle (79, 80), and some filamentous heterocystous cyanobacteria live in symbiosis with diatoms, providing them with fixed N_2_ (81, 82). Although *Coleofasciculaceae* ASVs could not be resolved to the species level, it is reported that members of this family are non-heterocystous (83), indicating that the strong presence of these cyanobacteria may not be *directly* linked to nitrogen fixation. Terrestrial members of the family *Coleofasciculaceae* are filamentous bundle-forming cyanobacteria, which often serve as keystone or pioneer species that facilitate further colonization of other organisms and establish healthy soil communities (83–86). These bundles provide space for microbial succession and colonization that become areas where diazotrophs can flourish (85). There is little to no information on *Coleofasciculaceae* in aquatic/marine systems, so this reasoning is speculative, but this report offers an opportunity for further investigation.

Broad taxonomic profiles of microbiomes provide a wealth of general information about diversity but do not provide information about niche differentiation among dominating taxa, like *Rhodobacteraceae* and *Coleofasciculaceae*. We conducted differential abundance analysis to identify specific ASVs that respond to the presence of the diatom (Fig. 4; Tables S1to S3). *Coleofasciculaceae* and *Rhodobacteraceae* ASVs were among the most differentially abundant across F/2 and SW yet did not have many common ASVs between the two media. This disparity of differentially abundant ASVs between F/2 and SW incubations once again highlights the need to study microbiomes in a medium that is as close to natural conditions as possible. The prevailing standard is to maintain phytoplankton cultures and their microbiomes in nutrient-supplemented media, which may not be conducive to unbiased microbiome investigations. There is likely a greater need for complex microbial associations in oligotrophic SW media as opposed to nutrient-rich F/2 to compensate for the nutrient supply either through competition (87) or mutualism (88). For example, ASVs with decreased differential abundance in SW may represent those taxa that require vitamins, which are not provided in SW. Unique ASVs belonging to the family *Rhodobacteraceae* were found to have both increased and decreased relative abundances in F/2 and SW (Table S1) that illustrate possible niche differentiation among closely related taxonomic members that prefer either to associate with phytoplankton or to be generalists (73).

### Toward a deeper understanding of early microbiome assembly

In order to identify specific *Coleofasciculaceae* and *Rhodobacteraceae* ASVs of importance, we constructed co-occurrence networks and identified particular nodes as hubs (Fig. S4 and S5; Data S1 to S3). The hubs correspond to keystone taxa that may be particularly important in structuring the microbial community (40). Nutrient levels also play a key role in shaping the microbiome, with nutrient-rich F/2 and nutrient-poor SW co-cultures showing different hub species. *Rhodobacteraceae* thrive and dominate as hubs in F/2 due to its abundance of nutrients, while SW’s scarcity leads to a more diverse taxonomic hub profile (Data S2 and S3). The presence of vitamins and micronutrients in F/2 can reduce the need for competition and mutualism among microbial communities (89). The differences in node connectivity suggest that under co-culture conditions, there are considerable changes in the way bacteria interact with each other. Differential association networks show that many more positive associations developed in SW co-cultures compared to F/2 co-cultures (Fig. S6 and S7) while topological differences and dissimilarity in network centrality measures indicate significant differences between co-culture and control networks (Data S2 and S3). The metabolic profile of the diatom is likely different under the oligotrophic conditions of SW, which may influence the production, accumulation, and release of secondary metabolites (90–92) and can directly or indirectly influence microbial interactions, which can ultimately modulate the microbiome.

The differential association network analysis in SW revealed that a number of positive associations developed between *Rhodobacteraceae* ASVs and ASVs classified as *Pelagibacter* and *Synechococcus* (Fig. S6 and S7). Members of the genera *Pelagibacter* and *Synechococcus* are numerically abundant in the open ocean and are known to possess streamlined genomes (93). Their reduced genomes mean that these free-living organisms may be dependent on co-occurring members of the microbiome for lost metabolic functions (94). Keystone species typically have a disproportionately large effect on the environment relative to their abundance. A number of computational, observational, and experimental studies allude to microbial communities containing keystone species, which are highly connected taxa that have considerable influence over microbiome architecture and function, irrespective of their relative abundance across spatial or temporal scales (95). Keystone species may achieve this by the indirect modulation of the wider community and/or through the production and utilization of metabolites (58, 96).

The pattern we observe in SW co-cultures is that beneficiaries (*Pelagibacter* and *Synechococcus*) develop positive associations with *Rhodobacteraceae* ASVs, many of which are classified as *Sulfitobacter* spp. (Fig. S7). The fact that *Sulfitobacter* species had a negative association with *Pelagibacter* and *Synechococcus* in the control suggests that *A. glacialis* might have promoted their growth in the co-culture, leading to their role as important taxa (possibly keystone taxa) in the developing microbiome by establishing a complex network of interactions. The complex association network observed in SW (Fig. 6) is likely the result of a requirement of taxa to interact in a truly oligotrophic environment, particularly forming associations between keystone *Sulfitobacter* ASVs and members with reduced genomes. These *Sulfitobacter* ASVs were examined in a broader context by placing the ASVs within a 16S rRNA phylogenetic tree of related *Rhodobacteraceae*, many of which are known symbionts of phytoplankton. The *Sulfitobacter* ASVs identified in this study were shown to be closely related to *Sulfitobacter* spp. that are symbionts of *Emiliania huxleyi* that offer protection against pathogenic bacteria (Fig. S8) (48). The importance of *Rhodobacteraceae* and *Sulfitobacter* spp. for our model diatom species is supported by previous studies where Behringer et al. (34) reported that *Rhodobacteraceae* make up a significant proportion of the *A. glacialis* microbiome, and a subsequent study by Shibl et al. (30) showed that *A. glacialis* promotes the growth of symbiotic *Rhodobacteraceae* isolated from its microbiome, specifically *Pseudosulfitobacter pseudonitzschiae* F5 (previously *Sulfitobacter pseudonitzschiae* F5) and *Phycobacter azelaicus* F10 (previously *Phaeobacter* sp. F10) over the opportunist, *Alteromonas macleodii* F12. These positive interactions between diatoms and *Sulfitobacter* spp. are not unique to *A. glacialis*; the diatom *Pseudonitzschia multiseries* has been shown to interact closely with *Sulfitobacter* SA11, supplying the bacterium with tryptophan, which it metabolizes into the hormone indole-3-acetic acid, which promotes diatom cell division (46). Similarly, *S. pseudonitzschiae* strain SMR1 was reported to stimulate the growth of its diatom partner, *Skeletonema marinoi* (47).

The interaction between members of the microbiome and its host ultimately plays an integral role in modulating the microbiome. Though there have not been reports of microbiome modulation by *Sulfitobacter* spp., members of the *Rhodobacteraceae* are known to bring about the modulation of the microbiome of marine eukaryotic hosts (97, 98). At this point, the exact mechanism by which *Sulfitobacter* spp. modulates the microbiome is yet to be elucidated. Genomic surveys of metagenome-assembled genomes closely related to *Sulfitobacter* spp. may offer some insight; it has been reported that *Sulfitobacter* spp. have the genetic potential to produce a number of secondary metabolites such as bacteriocins and polyketides, which could be involved in microbiome modulation (73). Taken together, there is precedence to support the notion that *Sulfitobacter* spp. may play an important role in the structuring of the diatom microbiome in this study.

### Conclusion

Microbiomes play pivotal roles in host health, development, and function. A number of studies over the years have brought to light the specificity and consistency with regard to bacterial taxa found in diatom and other phytoplankton microbiomes. Here, we examine the microbiome structuring of the diatom *Asterionellopsis glacialis* under eutrophic and oligotrophic conditions in the laboratory. There are caveats that this study does not address, which should be investigated in the future, the first being the effect that prefiltration might have on the development of the microbiome, as the removal of taxa that are attached to eukaryotic cells that could modulate the microbiome is not present in the inoculum. Secondly, environmental communities collected at different times and from different geographic locations might also influence the way the microbiome develops. Finally, there are a number of pitfalls associated with microbial network inferences, which include computational challenges such as the handling of data with a large number of zeros and randomization procedures to faulty predictions of relationships between two taxa that might have been indirectly influenced by a third; thus, great care needs to be undertaken when interpreting and drawing conclusions from them (99). However, our analysis and interpretation align with other experimental and computational studies (29, 30, 34, 73) regarding the importance of *Sulfitobacter* spp. to diatoms in general and *A. glacialis* in specifics. Our findings advance our understanding of phytoplankton microbiome assembly. Classical theories of community assembly do not take into consideration host effects while current investigations on phytoplankton microbiomes focus too heavily on host effects. To this end, this work takes the first steps toward a holistic explanation of phytoplankton microbiome assembly, one that takes into consideration niche differentiation, host filtering, and microbial network structuring.

## Data Availability

16S rRNA gene amplicon sequencing raw reads are deposited in NCBI under the BioProject accession number PRJNA1021337.

## SUPPLEMENTAL MATERIAL

Supplemental figures and data aem.00570-24-s0001.docx Fig. S1 to S8; Data S1 to S3.

Supplemental tables aem.00570-24-s0002.xlsx Tables S1 to S3.

ASM does not own the copyrights to Supplemental Material that may be linked to, or accessed through, an article. The authors have granted ASM a non-exclusive, world-wide license to publish the Supplemental Material files. Please contact the corresponding author directly for reuse.
