# Supplementary material for: Rhodobacteraceae are key players in microbiome assembly of the diatom Asterionellopsis glacialis
Source: Appl Environ Microbiol. 2024 May 29;90(6):e00570-24. doi: 10.1128/aem.00570-24 (PMC11218658; doi:10.1128/aem.00570-24)

***Supplementary Materials***

# Supplementary Tables (Please refer to Excel sheet)

# Supplementary Table 1: ASVs identified as differentially abundant for each day and for each culture medium with MetagenomeSeq (*p* adjusted < 0.05). Values of ef_logFC (i.e. Log_2_-fold change) highlighted in green indicates ASVs that have an increased abundance in the co-culture relative to the control for a given day.

**Supplementary Table 2:** Counts of differentially abundant ASVs per media type and across different days.

**Supplementary Table 3:** Taxonomic distribution of differentially increased and differentially decreased taxa per media type

# Supplementary Figures

**
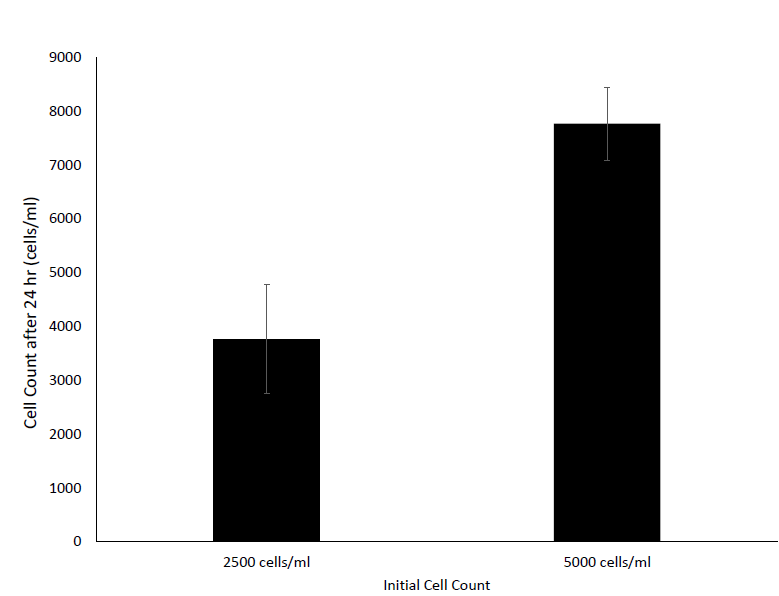
**

**Supplementary Figure 1.** Sustained growth of *A. glacialis* A3 in sterile seawater over a 24-hr period. *A. glacialis* A3 was inoculated at cell densities of ~10^3^ into sterile seawater and cell number counted microscopically after 24-hrs.


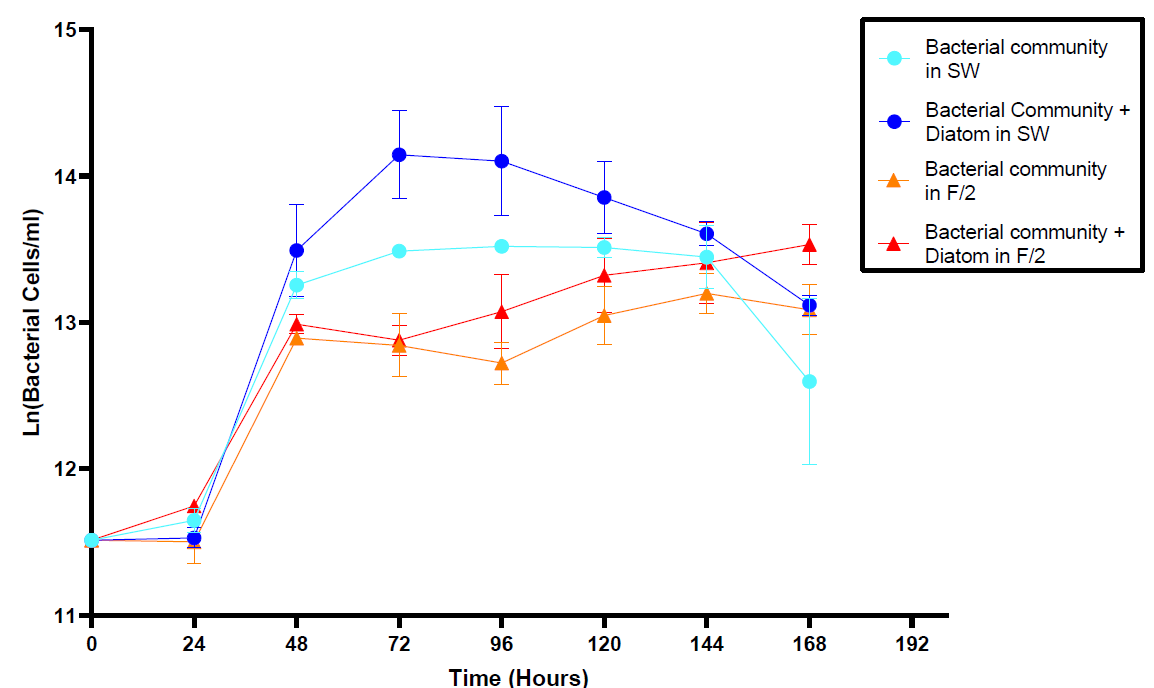


**Supplementary Figure 2.** Bacterial cell counts over the course of the seven-day incubation period in SW and F/2, with and without the diatom *A. glacialis* A3. Each day, a small volume was taken from each incubation and diluted in either SW or F/2 to keep a steady-state diatom cell density. Error bars represent the standard error.


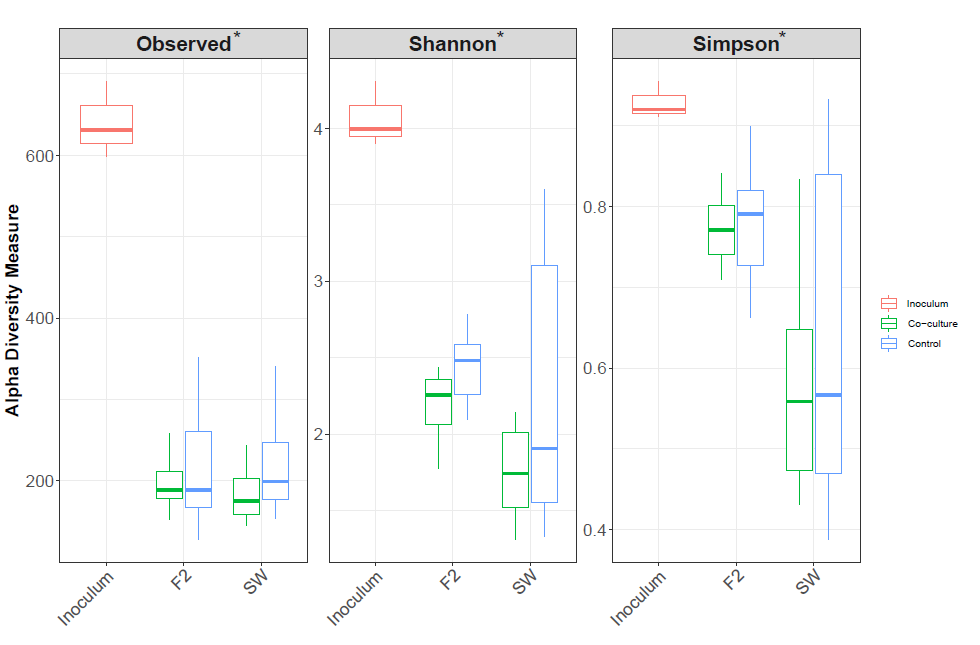


**a**


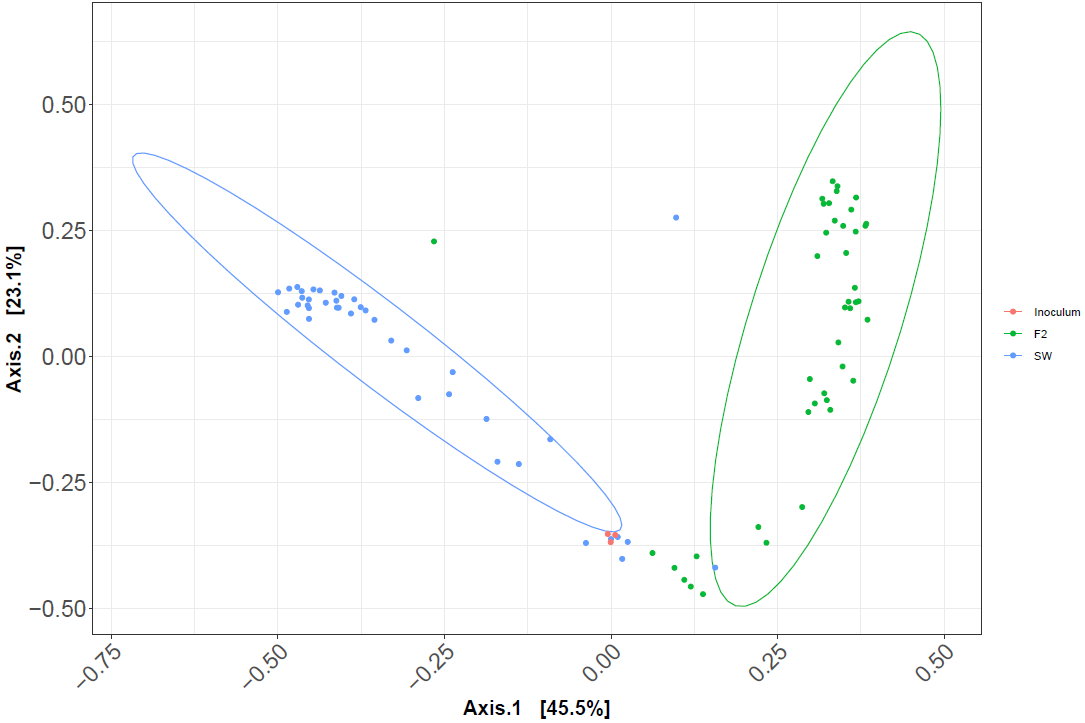


**b**

**Supplementary Figure 3.** Microbiome diversity in F/2 and SW. **a**. Alpha-diversity indices of observed ASVs, Shannon and Simpson of starter inoculum, controls and co-cultures for each culture medium. *indicates significant differences observed between inoculum and F2 and SW incubations for the respective α-diversity indices (Wilcox, *p* < 0.01, *p* < 0.01 and *p* < 0.05 for Observed, Shannon and Simpson indices, respectively). **b.** PCoA of Bray-Curtis distances between starter inoculum and culture incubations (PERMANOVA, *p* < 0.001).

**
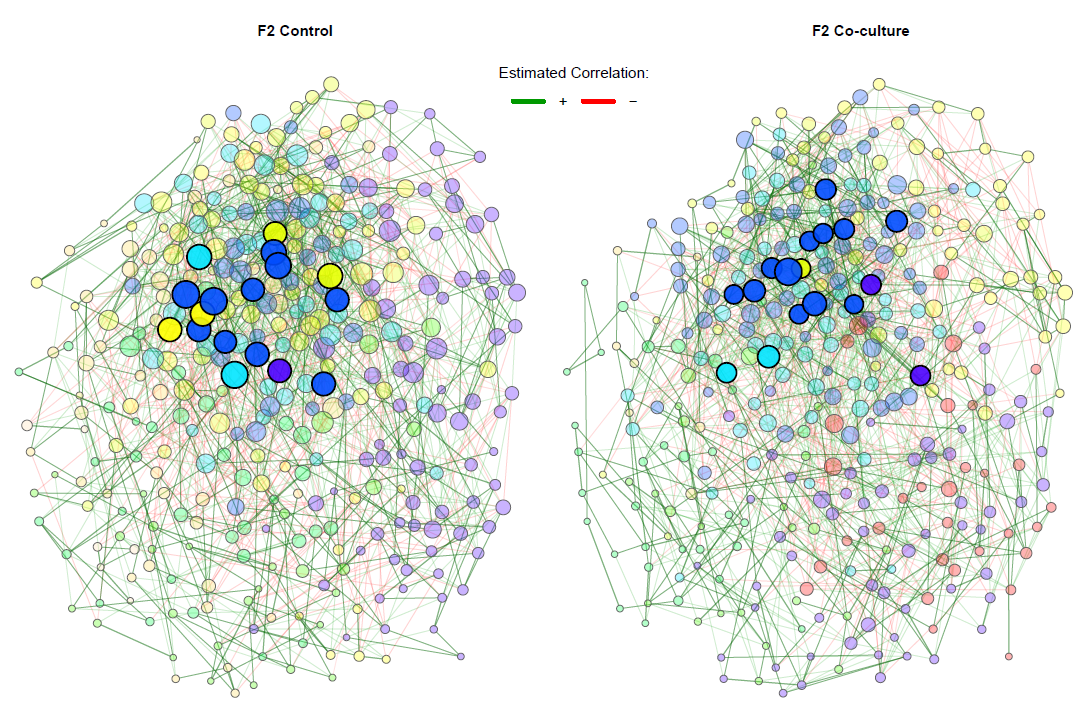
**

**Supplementary Figure 4.** Global co-occurrence network of bacterial taxa in control and co-culture incubations in F/2 media. Nodes represent individual ASVs while edges represent either positive (green) or negative (red) associations. Nodes that share the same color form a cluster that is more connected relative to other nodes. The size of the node is relative to the eigenvector centrality value, i.e. the larger the node, the more central that node is to the network as a whole. Nodes that are less-transparent and that have dark outlines have been identified as hubs, based on their eigenvector centrality. Global network features and results for the quantitative analysis can be found in Supplementary Table 2.

**
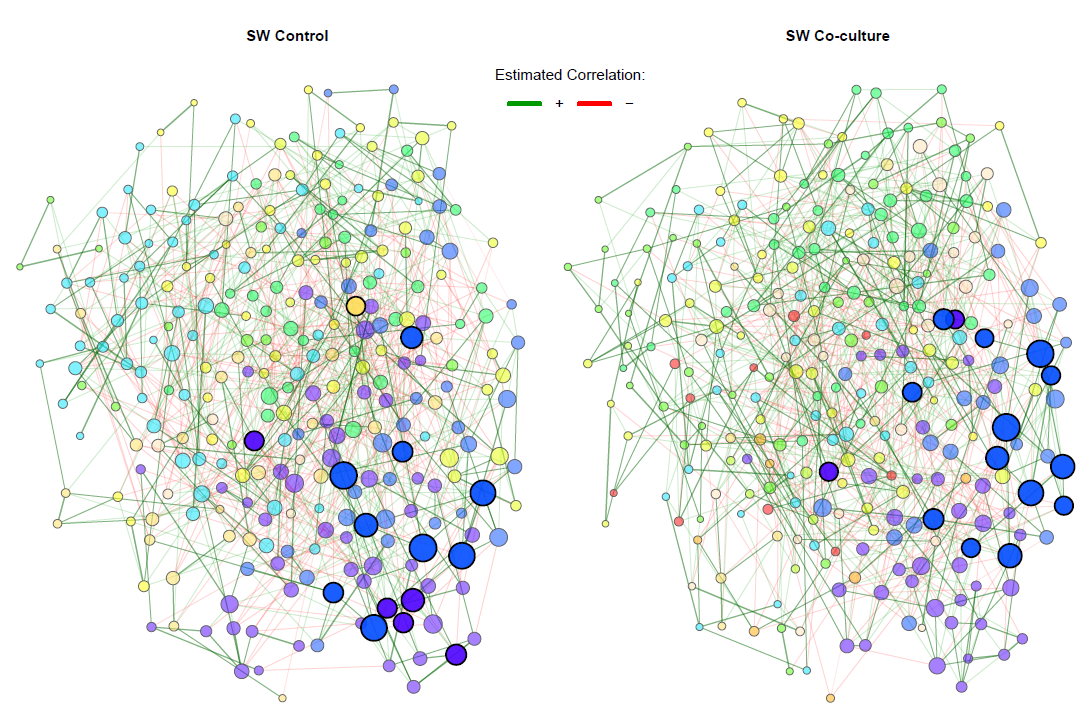
**

**Supplementary Figure 5.** Global co-occurrence network of bacterial taxa in control and co-culture incubations in SW media. Nodes represent individual ASVs while edges represent either positive (green) or negative (red) associations. Nodes that share the same color, form a cluster that is more connected relative to other nodes. The size of the node is relative to the eigenvector centrality, i.e. the larger the node, the more central that node is to the network as a whole. Nodes that are less-transparent and that have dark outlines have been identified as hubs, based on their eigenvector centrality. Global network features can be found in Supplementary Data 1.

**
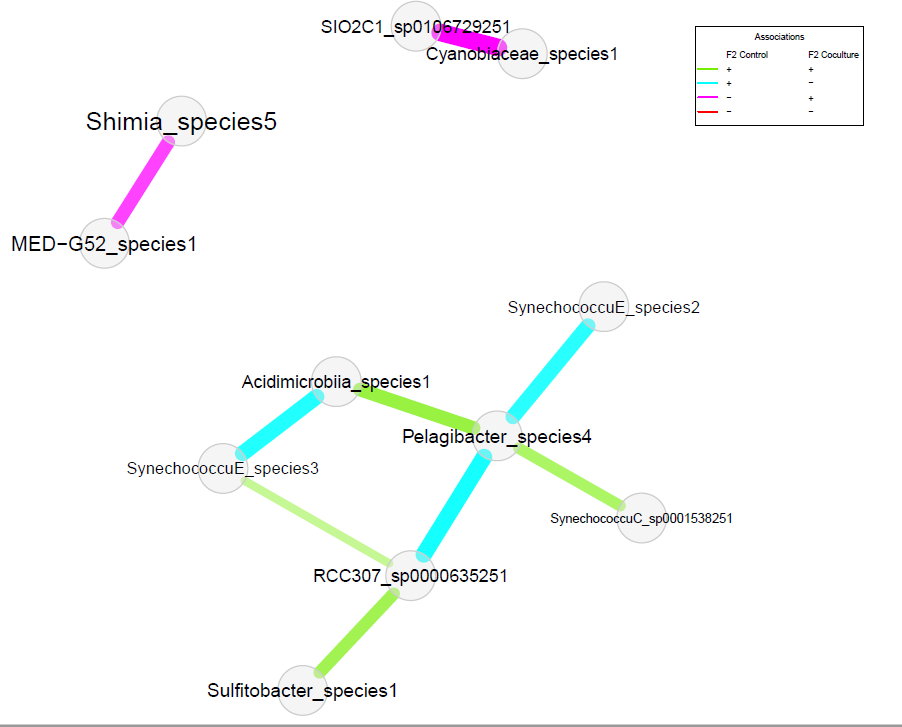
**

**Supplementary Figure 6.** Differential association network for F/2 incubations. Spearman correlations were created and significantly differentially associated taxa were identified by comparing the correlation coefficients with Fisher’s z-test (default *lfdr* <= 0.2) (Peschel *et al*., 2021). Nodes represent individual ASVs and edge color represents differential associations between control and co-culture networks. Edge width represents strength of the association with thicker lines indicating stronger associations. Quantitative analysis results can be found in Supplementary Data 2.

**
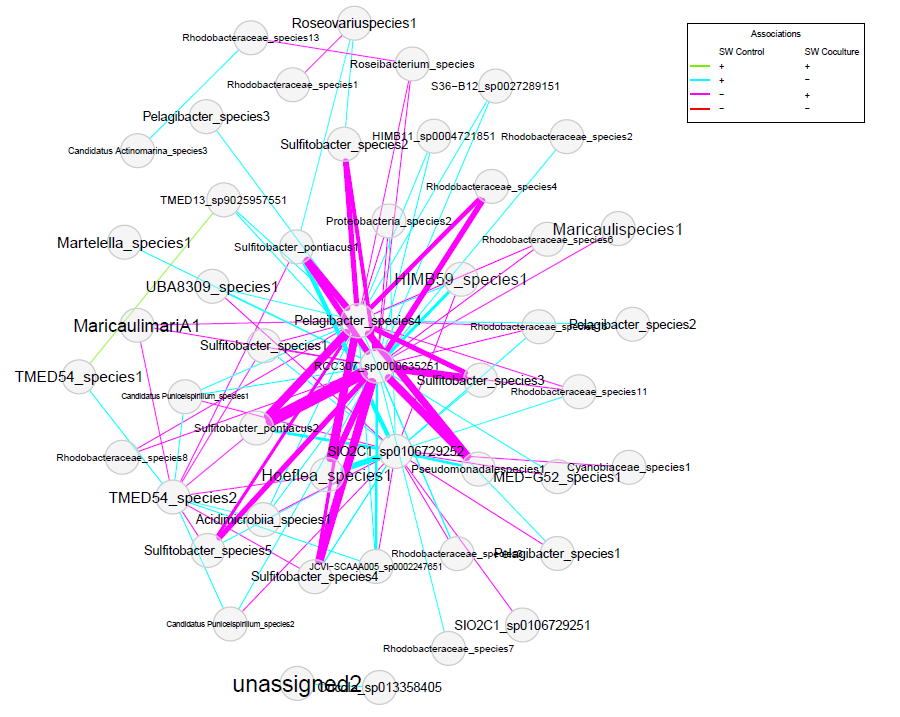
**

**Supplementary Figure 7.** Differential association network for SW incubations. Spearman correlations were created and significantly differentially associated taxa were identified by comparing the correlation coefficients with Fisher’s z-test (default *lfdr* <= 0.2) (Peschel *et al*., 2021). Nodes represent individual ASVs and edge color represents differential associations between control and co-culture networks. Edge width represents strength of the association with thicker lines indicating stronger associations. Quantitative analysis results can be found in Supplementary Data 3.


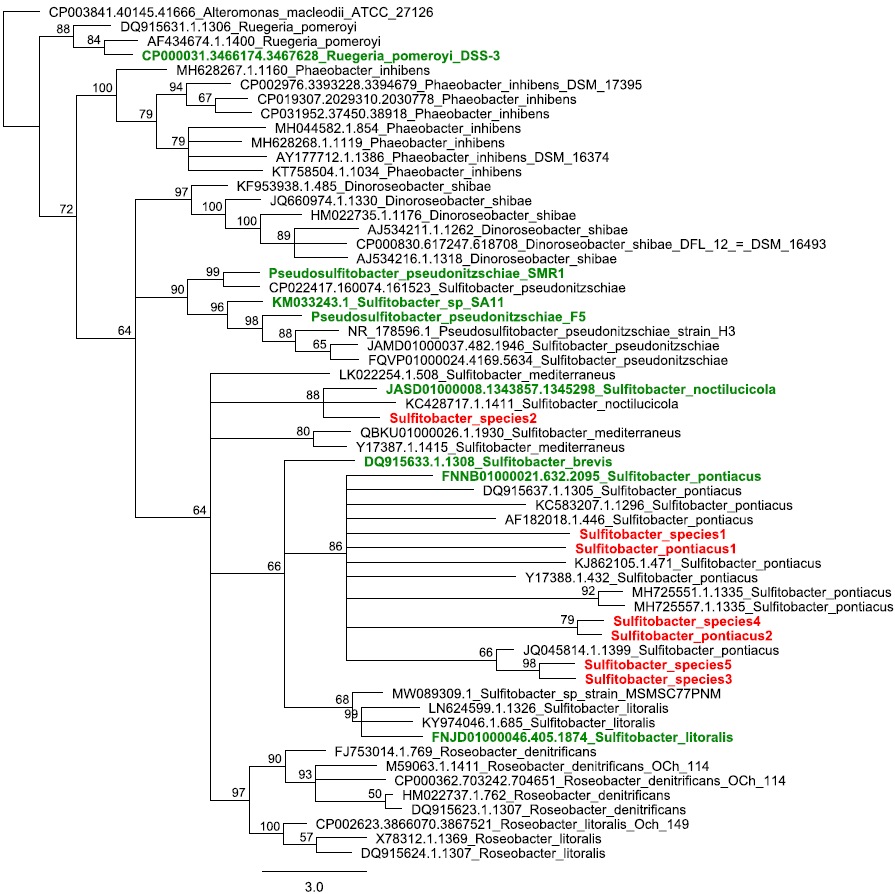


**Supplementary Figure 8.** Maximum likelihood phylogenetic tree generated from *Sulfitobacter* ASVs (highlighted in red) and 16S rRNA genes from diverse members of the family *Rhodobacteraceae.* Node supports are bootstrap values of 100 replicates. Taxa highlighted in green are isolates that are known to be symbionts of phytoplankton. Leaf labels represent SILVA and NCBI accession numbers followed by scientific names of the organism.

# Supplementary Data

**Supplementary Data 1:** Global co-occurrence network properties of F/2 and SW microbiomes and quantitative analysis of control and co-culture networks for each culture medium. In each case, ‘group 1’ represents the control and ‘group 2’ represents the co-culture. The Global Network Properties panel provides descriptive information of the sparse networks such as size (number of nodes) and positive edge connectivity.


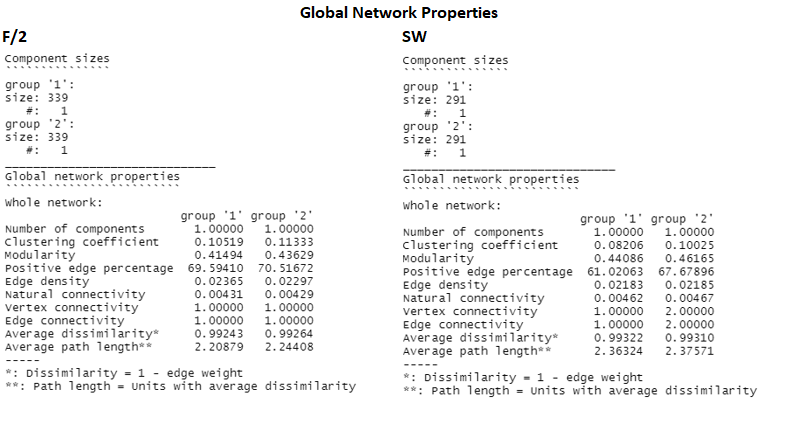


**Supplementary Data 2:** Quantitative information by comparing control (‘group 1’) and co-culture (‘group 2’) networks for F/2. Panels include hub taxa and top 5 ASVs with the highest eigenvector centrality for each condition. Additionally, Jaccard indices and Adjusted Rand indices are provided, which provide information on the similarity of nodes and centrality measures, and clustering similarity, respectively.


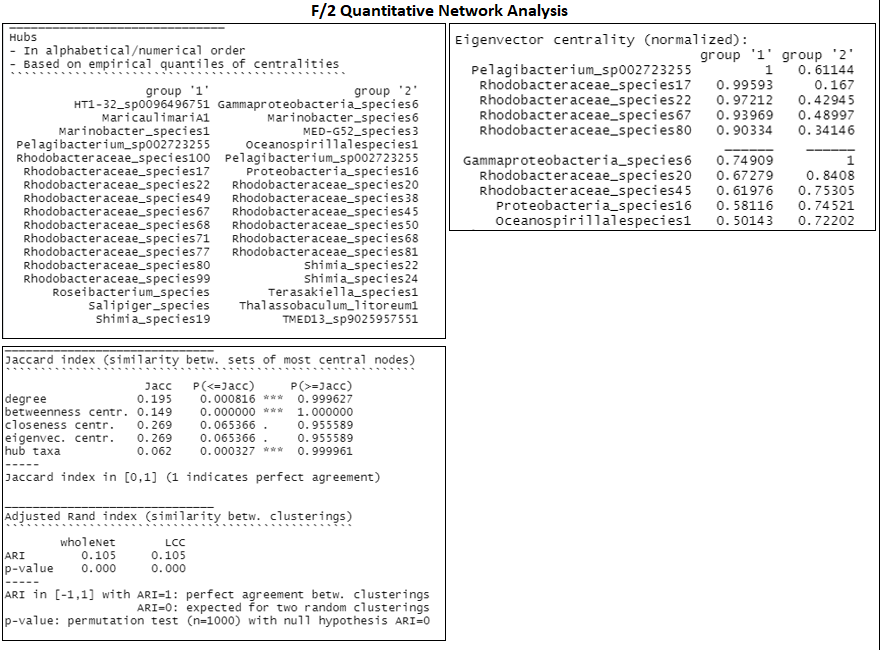


**Supplementary Data 3:** Quantitative information by comparing control (‘group 1’) and co-culture (‘group 2’) networks for SW. Panels include hub taxa and top 5 ASVs with the highest eigenvector centrality for each condition. Additionally, Jaccard indices and Adjusted Rand indices are provided, which provide information on the similarity of nodes and centrality measures, and clustering similarity, respectively.


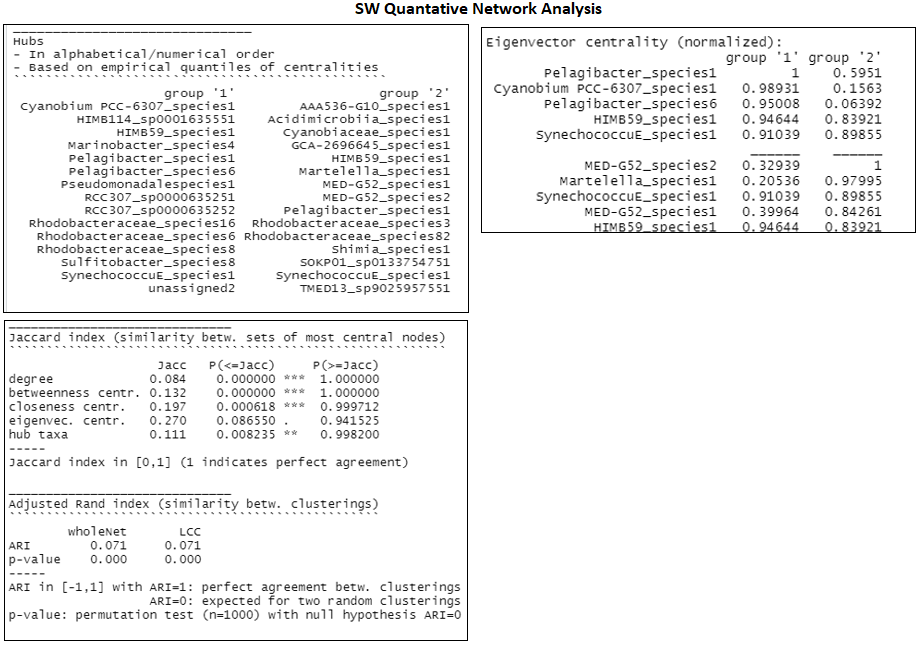

Supplement: Supplemental figures and data — Fig. S1 to S8; Data S1 to S3. [file aem.00570-24-s0001.docx]
